# Extracellular Vesicles in Reproduction: Biology, Production, and Potential Applications in Livestock Breeding

**DOI:** 10.1111/rda.70112

**Published:** 2025-09-03

**Authors:** Alireza Fazeli, Kasun Godakumara, Suranga Kodithuwakku, Subhashini Muhandiram

**Affiliations:** ^1^ Institute of Veterinary Medicine and Animal Sciences Estonian University of Life Sciences Tartu Estonia; ^2^ Division of Clinical Medicine School of Medicine & Population Health, University of Sheffield Sheffield UK; ^3^ Department of Pathophysiology Institute of Biomedicine and Translational Medicine, University of Tartu Tartu Estonia; ^4^ Department of Animal Science, Faculty of Agriculture University of Peradeniya Peradeniya Sri Lanka

**Keywords:** diagnostics, extracellular vesicles, fertility, livestock, reproductive biology, therapeutics

## Abstract

Extracellular Vesicles (EVs) are small, membrane‐bound particles released by cells into biological fluids, where they function as mediators of intercellular communication. These vesicles transport a diverse array of bioactive molecules, including proteins, lipids, and nucleic acids, and play essential roles in regulating physiological and pathological processes. Recent research has revealed the significance of EVs in reproductive biology, particularly in the areas of spermatozoa maturation, oocyte development, embryo implantation, and maternal‐fetal interactions. Given their widespread distribution and biological importance, EVs have been increasingly studied for their potential applications in both human and livestock reproductive medicine. Understanding the mechanisms by which EVs contribute to reproductive processes is crucial, as they offer novel opportunities for improving reproductive health, diagnosing fertility disorders, and enhancing assisted reproductive technologies. In males, EVs derived from seminal plasma and the epididymis influence sperm motility, capacitation, and fertilisation potential. In females, vesicles secreted within follicular, oviductal, and uterine fluids mediate communication between the oocyte, embryo, and maternal reproductive tract. Furthermore, placental‐derived EVs regulate immune tolerance, vascular remodelling, and fetal development throughout pregnancy. EVs are emerging as promising tools for fertility assessment and reproductive diagnostics. Their molecular cargo reflects the physiological state of the reproductive system, enabling their use as non‐invasive biomarkers for evaluating gamete quality, embryo viability, and pregnancy health. Despite their immense potential, challenges remain in optimising EV isolation, improving characterisation techniques, and deciphering the precise molecular mechanisms underlying their function. Standardisation of methodologies, development of targeted vesicle‐based therapeutics, and validation of their efficacy in reproductive medicine are necessary to fully realise their clinical utility. The field of EV research in reproductive biology continues to evolve rapidly, and ongoing studies will undoubtedly lead to new insights into their role in fertility, embryo development, and pregnancy maintenance.

## 1 Introduction

Extracellular Vesicles (EVs) are widely recognised as key mediators of intercellular communication and have been identified in virtually all biological fluids (Lättekivi et al. 2022; Rodriguez‐martinez and Roca 2022; Sun and Lerman 2020; Van Herwijnen et al. 2016). These vesicles, which range in size from 30 to 1000 nm, serve as carriers of bioactive molecules that regulate various physiological processes. Their presence in reproductive fluids, including seminal plasma (Reshi et al. 2021), follicular fluid (Hasan et al. 2021), oviductal fluid (Almiñana et al. 2017), and uterine secretions (Piibor et al. 2023), has sparked significant interest for studying their involvement and function in reproductive biology and physiology.

Reproduction is a highly complex process that involves intricate signalling between gametes, the reproductive tract (Ghersevich et al. 2015; Hasan et al. 2021), and the developing embryo (Guzewska et al. 2023; Muhandiram et al. 2023; Segura‐Benítez et al. 2022). The discovery that EVs facilitate molecular crosstalk at multiple stages of reproduction has led to increasing investigations into their biological significance (Fazeli and Godakumara 2024). In the male reproductive system, EVs may influence spermatozoa maturation and development, capacitation (Hasan et al. 2021) and interactions of spermatozoa with the female reproductive tract (Reshi et al. 2023). In females, EVs secreted in the follicular and endometrial environment contribute to oocyte competence (Makieva, Saenz‐de‐Juano, et al. 2024), embryo implantation (Evans et al. 2019; Muhandiram et al. 2023), and maternal immune modulation during pregnancy (Wu et al. 2022).

The study of EVs in reproductive medicine has expanded due to their potential as biomarkers for fertility assessment (Dissanayake et al. 2021; Rana et al. 2024) and potential applications for diagnosing reproductive disorders (Muraoka et al. 2024a; Piibor et al. 2024). Their cargo composition, which includes proteins, microRNAs, and lipids, may reflect the physiological status of the reproductive system (Hart et al. 2023; Mousavi et al. 2024), making them ideal candidates for non‐invasive diagnostics. Additionally, EV‐based therapies are being explored for enhancing in vitro fertilisation outcomes (Franko and de Almeida Monteiro Melo Ferraz 2024), improving embryo culture conditions (Bauersachs et al. 2020), and even developing novel treatments for infertility (Liu et al. 2020; Poh et al. 2023).

The recognition of EVs as central players in reproductive processes has prompted further research into their biogenesis, molecular function, and translational applications. However, challenges such as standardising isolation techniques, characterising heterogeneous vesicle populations, and elucidating their precise functional roles remain significant obstacles in the further development of the field. Continued advancements in molecular biology, nanotechnology, and reproductive sciences will be essential for fully understanding the function of EVs in any physiological system, including reproductive systems. In addition, such developments will allow harnessing the therapeutic and diagnostic potential of EVs in reproductive medicine.

## 2 Biology and Biogenesis of EVs

EVs are categorised based on their size, mode of biogenesis, and functional properties. They include exosomes, which are the smallest vesicles ranging from 30 to 150 nm, microvesicles, which are larger and typically range from 150 to 1000 nm, and apoptotic bodies, which can reach up to 2000 nm in size (Welsh et al. 2024; Yáñez‐Mó et al. 2015). These vesicles are released into the extracellular environment through distinct cellular mechanisms, each contributing to their unique molecular composition and function (Dissanayake et al. 2024; Hagey et al. 2023).

Exosomes originate from endosomes; they form endosomal compartments that contain intraluminal vesicles. These vesicles are released into the extracellular space through fusion of multivesicular bodies with the plasma membrane (Kalluri and LeBleu 2020). Microvesicles, on the other hand, are formed by outward budding of the plasma membrane, a process regulated by lipid reorganisation and cytoskeletal remodelling (Tricarico et al. 2017). Apoptotic bodies are generated during programmed cell death and contain remnants of cellular components, including nuclear fragments, organelles, and cytoplasmic proteins (Battistelli and Falcieri 2020).

The cargo of EVs is selectively sorted and loaded into vesicles through complex regulatory pathways (Lee et al. 2024). The endosomal sorting complex required for transport, also known as ESCRT, is a major determinant of exosome biogenesis and is responsible for packaging specific biomolecules into vesicles (Frankel and Audhya 2018). Lipid raft‐associated pathways (De Gassart et al. 2003) and tetraspanin proteins also play key roles in vesicle cargo selection. The composition of EVs is highly dynamic and varies depending on the cell of origin (Hagey et al. 2023), physiological conditions (Hart et al. 2023), and environmental stimuli (Mousavi et al. 2024).

EVs are enriched in proteins such as heat shock proteins, tetraspanins, and integrins, which are involved in cell adhesion, signalling, and stress responses. Lipid analysis of vesicle membranes has revealed a unique composition of sphingolipids, ceramides, and cholesterol, contributing to membrane stability and fusion properties (Ghadami and Dellinger 2023; Haraszti et al. 2016). The presence of microRNAs, messenger RNAs, and long non‐coding RNAs within vesicles further highlights their role in post‐transcriptional gene regulation (O'Brien et al. 2020). The ability of EVs to transfer functional nucleic acids between cells has profound implications for reproductive biology, as they can modulate gene expression and cellular behaviour in target tissues (Dissanayake et al. 2024; Es‐Haghi et al. 2019).

Advances in omics technologies, including proteomics, lipidomics, and transcriptomics, have greatly enhanced our understanding of EVs composition (Blandin et al. 2023; Ghanam et al. 2022; Hayasaka et al. 2023; Lischnig et al. 2022). High‐throughput sequencing and mass spectrometry have allowed researchers to identify key molecular signatures associated with reproductive EVs. These insights are essential for deciphering the functional roles of vesicles in gamete development, fertilisation, and embryo implantation (Beal et al. 2023; Mazzarella et al. 2024; Piibor et al. 2023, 2024).

Despite significant progress, challenges remain in isolating and characterising EVs with high specificity. Current isolation methods, such as ultracentrifugation, size‐exclusion chromatography, and immunoaffinity capture, each have limitations in terms of purity, yield, and vesicle integrity (Brennan et al. 2020; Welsh et al. 2024). The development of microfluidic‐based platforms and single‐vesicle analysis techniques will be crucial for improving EV isolation and characterisation (Gao et al. 2023).

The study of EV biogenesis has provided important insights into their regulatory mechanisms and functional relevance (Dar et al. 2021; Dixson et al. 2024). Understanding how vesicles are formed, packaged, and secreted will pave the way for developing targeted therapeutic strategies that exploit their natural signalling capabilities (Hadizadeh et al. 2022). Given their potential for modulating reproductive processes, EVs represent a promising avenue for advancing fertility research and clinical applications (Parvin et al. 2024).

## 3 Role of EVs in Male Fertility

EVs play a crucial role in male reproductive physiology, particularly in the processes of sperm maturation, motility regulation, and fertilisation (Rana et al. 2024; Xu et al. 2024). The male reproductive tract is composed of several distinct regions, including the testes, epididymis, prostate, and seminal vesicles, all of which contribute secretions that ultimately form the seminal plasma (Perumal 2012; Rodriguez‐Martinez et al. 2021). The fluid component of semen (seminal plasma) contains a complex mixture of molecules, including proteins, hormones, lipids, and EVs (Evans et al. 2021; Jodar et al. 2016; Wang et al. 2022). These vesicles act as carriers of regulatory molecules that influence sperm physiology and fertilisation capacity.

Spermatogenesis, the process of sperm cell production, occurs within the seminiferous tubules of the testes and involves a complex series of cell divisions and differentiation steps (Nishimura and L'Hernault 2017). The spermatozoa that emerge from the testes are structurally complete but functionally immature (Schubert 2016). They acquire motility and fertilisation potential during their transit through the epididymis, a highly specialised ductal system where epididymal EVs contribute to post‐testicular sperm maturation (Gervasi and Visconti 2017). Epididymosomes, a specific subset of EVs found in the epididymal lumen of the epididymis, have been shown to transfer proteins, lipids, and non‐coding RNAs to spermatozoa, modulating their membrane composition and functional properties (Ali et al. 2023; Barrachina et al. 2022).

EVs also regulate sperm motility by influencing ion channel activity and the metabolic state of spermatozoa (Pinto et al. 2023). The acquisition of motility is essential for spermatozoa to reach and penetrate the oocyte, and EVs in seminal plasma contain signalling molecules that enhance sperm energetics and cytoskeletal rearrangements required for progressive motility (Han, Li, et al. 2024; Tamessar et al. 2024; Zhang, Liang, et al. 2024). Additionally, capacitation, a process that prepares spermatozoa for the acrosome reaction and fertilisation (Xu et al. 2024), is influenced by EVs containing cholesterol efflux regulators and enzymes that modify sperm membrane fluidity (Hasan et al. 2021; Travis and Kopf 2002).

The role of EVs extends beyond sperm motility and capacitation to sperm–egg recognition and interaction. During fertilisation, spermatozoa must first penetrate the cumulus cell layers surrounding the oocyte before binding to the zona pellucida, an extracellular matrix that encases the egg (Lange‐Consiglio et al. 2022). Sperm‐derived EVs have been implicated in facilitating this interaction by transferring zona pellucida‐binding proteins and proteases that help in zona penetration (Pal et al. 2025; Wang et al. 2022). Furthermore, seminal plasma EVs also modulate the immune response of the female reproductive tract to ensure sperm survival and tolerogenic immune conditions for successful fertilisation (Zhang, Greve, et al. 2024).

Emerging studies suggest that defects in EV‐mediated signalling can lead to male infertility (Parra et al. 2023; Xu et al. 2024). Aberrant composition of EVs in seminal plasma has been associated with impaired sperm motility, increased oxidative stress, and decreased fertilisation potential (Cannarella et al. 2020; Han, Li, et al. 2024). The use of EV biomarkers for assessing spermatozoa quality and diagnosing male infertility is a growing area of interest, offering a non‐invasive means of evaluating reproductive potential (Rana et al. 2024).

## 4 Role of EVs in Female Fertility

EVs are also critical in female reproductive processes, particularly in oocyte maturation, follicular development, and embryo implantation (Machtinger et al. 2021). The female reproductive tract is a highly dynamic environment that undergoes cyclical changes regulated by hormonal fluctuations (Hawkins and Matzuk 2008). EVs mediate molecular communication within the follicular (Hasan et al. 2021), oviductal (Dissanayake et al. 2021), and uterine microenvironments (Godakumara et al. 2021; Piibor et al. 2024).

Follicular fluid, which surrounds the developing oocyte within the ovarian follicle, contains EVs secreted by granulosa cells, theca cells, and the oocyte itself (Lai et al. 2015). These vesicles play an essential role in oocyte competence, which refers to the ability of an oocyte to undergo successful fertilisation and embryonic development (Gabryś et al. 2022). The cargo of follicular EVs includes growth factors, cytokines, and microRNAs that regulate follicular cell proliferation, oocyte metabolic activity, and meiotic progression (Benedetti et al. 2024; Uzbekova et al. 2020). In particular, EVs contribute to the transfer of small RNAs involved in epigenetic modifications, which may influence oocyte developmental potential (Aoki et al. 2024; Martinez et al. 2018).

After ovulation, the oocyte enters the oviduct, where fertilisation occurs. The oviductal fluid provides a supportive environment for sperm capacitation, fertilisation, and early embryo development (Ferraz et al. 2019). EVs secreted by oviduct epithelial cells have been shown to facilitate sperm storage and survival within the oviduct by preventing premature capacitation (Alcântara‐Neto et al. 2020; Ferraz et al. 2019). These vesicles also contribute to spermatozoa selection by influencing the molecular composition of the oviductal reservoir where spermatozoa are retained before fertilisation (Lange‐Consiglio et al. 2022).

Following fertilisation, the early embryo undergoes a series of cleavage divisions while travelling through the oviduct toward the uterus. During this pre‐implantation period, EVs in the oviductal and uterine fluids provide essential signals that regulate embryo metabolism, gene expression, and immune tolerance (Poh et al. 2022; Segura‐ben et al. 2025). The transfer of maternal RNAs and proteins via EVs has been suggested to play a role in embryo quality and implantation success (Es‐Haghi et al. 2019; Leal et al. 2022).

## 5 Embryo‐Maternal Crosstalk Mediated by EVs

Successful pregnancy requires complex molecular communication between the developing embryo and the maternal endometrium. This process, known as embryo‐maternal crosstalk, is largely mediated by EVs, which serve as molecular messengers facilitating bidirectional signalling between the embryo and the uterine lining.

Prior to implantation, the blastocyst must establish a receptive environment within the uterus. EVs secreted by trophoblast cells, the outer layer of the blastocyst, interact with maternal immune cells and endometrial epithelial cells to promote endometrial receptivity (Godakumara et al. 2021; Godakumara et al. 2023; Makieva, Giacomini, et al. 2024; Muhandiram et al. 2023; Poh et al. 2021). These vesicles carry signalling molecules such as cytokines, integrins, and microRNAs that regulate endometrial remodelling and vascularisation, ensuring adequate blood supply to the implantation site (Fatmous et al. 2022; Guzewska et al. 2023; Poh et al. 2023).

EVs also play a crucial role in immune regulation during pregnancy. The maternal immune system must tolerate the presence of the semi‐allogeneic fetus while maintaining immune surveillance to prevent infections. Trophoblast‐derived EVs contribute to maternal immune tolerance by modulating the activity of immune cells, including T cells, macrophages, and natural killer cells (Favaro et al. 2021; Wu et al. 2024). These vesicles suppress inflammatory responses and promote an anti‐inflammatory environment conducive to fetal development.

Dysregulation of EV‐mediated communication during implantation has been implicated in pregnancy complications such as recurrent implantation failure and early pregnancy loss (Sun et al. 2025; Zhang et al. 2020). Abnormal EV cargo, altered secretion patterns, and disrupted vesicle uptake by maternal cells may contribute to implantation failure and placental dysfunction (Makieva, Giacomini, Giacomini, et al. 2024; Segura‐Benítez et al. 2022).

## 6 EVs in Placental Function and Fetal Development

The placenta is a vital organ that facilitates nutrient exchange, gas exchange, and immune modulation between the mother and fetus (Gude et al. 2004). EVs derived from placental trophoblasts have been identified in maternal circulation and play an essential role in pregnancy maintenance (Kupper and Huppertz 2022; Tong et al. 2018).

Placental EVs regulate vascular remodelling and angiogenesis by transferring pro‐angiogenic factors to endothelial cells (Cronqvist et al. 2020). These vesicles contain growth factors such as vascular endothelial growth factor (VEGF) and placental growth factor (PlGF), which stimulate the formation of new blood vessels within the maternal–fetal interface. Proper vascularisation of the placenta is critical for fetal oxygenation and nutrient delivery (Feng et al. 2022; Gebara et al. 2021).

EVs also influence metabolic adaptations during pregnancy. Placenta‐derived vesicles carry metabolic enzymes and transporters involved in glucose homeostasis, lipid metabolism, and fetal nutrient uptake. These vesicles ensure optimal fetal growth by modulating maternal metabolic pathways (Renaud et al. 2023; Rosenfeld 2024).

Pregnancy complications such as preeclampsia, gestational diabetes, and intrauterine growth restriction have been linked to altered EV profiles in maternal circulation (Levine et al. 2020; Ortega et al. 2022). Analysing the composition of placental EVs may provide valuable insights into pregnancy health and allow for early diagnosis of gestational disorders (Chaemsaithong et al. 2023).

## 7 Clinical Applications of EVs in Reproductive Medicine

The clinical applications of EVs in reproductive medicine are vast, with potential uses in fertility diagnostics (Muraoka et al. 2024b; Rana et al. 2024), ART enhancement (Fang et al. 2023), and therapeutic interventions (Xue et al. 2024). One of the most promising applications is the use of EVs as biomarkers for assessing fertility status. Clinicians may be able to predict sperm quality (Pal et al. 2025), oocyte competence (da Silveira et al. 2012), and embryo viability (Dissanayake et al. 2021; Es‐Haghi et al. 2019) by analysing the molecular composition of EVs in seminal plasma, follicular fluid, and uterine secretions.

In assisted reproductive technologies, EVs have been explored as tools for improving embryo culture conditions (Xue et al. 2024). Supplementing culture media with EVs derived from reproductive fluids may enhance embryo development by providing essential growth factors and protective molecules (Leal et al. 2022; Poh et al. 2023).

## 8 Applications of EVs in Livestock Breeding

EVs have significant potential in improving reproductive outcomes in livestock species such as cattle, pigs, sheep, goats, and horses breeding programmes (Table 1). The global livestock industry relies heavily on assisted reproductive technologies, including artificial insemination, embryo transfer, and in vitro fertilisation, to enhance genetic traits and reproductive efficiency (Gadea et al. 2020; Mikkola et al. 2024; Verma et al. 2012). Despite advancements in these technologies, fertility rates remain suboptimal due to limitations in sperm cryopreservation (Donnelly et al. 2001; Tanga et al. 2021), embryo viability (Erdem et al. 2020; Lopera‐Vasquez et al. 2017), and maternal receptivity (Binelli et al. 2022; Paulson and Comizzoli 2021). Emerging research suggests that EVs can provide novel solutions for overcoming these challenges by modulating sperm function (Mahdavinezhad et al. 2022), embryo–maternal communication (Hu et al. 2022; Xue et al. 2024), and pregnancy maintenance (Galli et al. 2024).

**TABLE 1:** Potential applications of EVs in livestock breeding.

Potential applications of EVs in livestock breeding	Species	In vitro/In vivo	Key findings	References
EVs as fertility biomarkers	Chicken	In vitro	Smaller EVs in seminal plasma appeared more abundant in fertile than in subfertile roosters. HSP90A was significantly more abundant in fertile than in subfertile males seminal plasma EVs. Co‐incubation seminal plasma EVs with sperm showed a higher capacity to be incorporated into fertile than into subfertile sperm. Sperm viability and motility were impacted by the presence of EV from fertile males	Cordeiro et al. (2018)
	Bovine	In vitro	EVs present in bovine follicular fluid of antral follicles of similar morphology contain lipids that may be used as biomarkers associated with the developmental capability of the oocyte to develop to the blastocyst stage	da Silveira et al. (2021)
	Chicken	In vitro	The seminal plasma EVs was successfully isolated from 4 different chicken breeds and miRNA was sequenced. Seminal plasma EV coupled miRNA have roles in sperm maturation and regulating the female's immune response and lipid metabolism, therefore have the potential to use as biomarkers of fertility	Han et al. (2023)
	Buffalo	In vitro	The proteome of seminal plasma exosomes differs between seminal plasma associated with high‐motility and low‐motility spermatozoa	Yu et al. (2023)
	Boars	In vitro	Seminal plasma EV‐derived miRNAs reflect boar sperm quality	Chen et al. (2025); Dlamini et al. (2023)
	Sahiwal cattle	In vitro	bta‐miR‐195 in seminal plasma EVs had 80% higher expression in high fertility bulls compared to low fertility bulls, suggesting its association fertility status	Chauhan et al. (2024)
	Stallions	In vitro	Particle size of seminal plasma EVs collected from good freezability ejaculates were different from poor freezability ejaculates	Barranco et al. (2025)
Role of EVs in enhancing sperm function and cryopreservation	Boar	In vitro	Pig prostasome‐like vesicles are able, in vitro, to interact with spermatozoa and to stimulate the acrosome reaction	Siciliano et al. (2008)
	Boar	In vitro	Adding boar seminal plasma exosomes to boar sperm preparations increased their functional parameters such as sperm motility, prolonged effective survival time, improved sperm plasma membrane integrity, increased total antioxidant capacity activity and decreased malondialdehyde content. This effect was dose dependent	Du et al. (2016)
	Bovine	In vitro	Follicular fluid derived EVs were able to modulate the viability, capacitation and acrosome reaction of bull spermatozoa	Hasan et al. (2021)
	Sahiwal cattle bulls	In vitro	Supplementing low fertility bull spermatozoa with high fertility bull seminal plasma EVs could enhance their functional characteristics	Pal et al. (2025)
	Bovine	In vitro	Oviductal fluid derived EVs carry sperm interacting proteins such as OVGP1, ACTB, HSP27, MYH9, MYH14 and OVGP1 and their abundance change across menstrual cycle. Therefore, above protein candidates in oviductal fluid were identified as modulating sperm functions	Lamy et al. (2004)
	Bovine	In vitro	EVs from bull semen plasma significantly improve cryostability of cells by supporting the potentials of the mitochondrial membrane and protecting the cytoplasmic membrane of spermatozoa	Kowalczyk and Kordan (2024)
	Stallion	In vitro	Equine mesenchymal stem cells (derived from adipose tissue) derived EVs enhances stallion sperm motility, progressive movement and viability	Sawicki et al. (2024)
EVs in improving oocyte maturation and embryo development	Bovine	In vitro	Exosomes in follicular fluid play important roles during oocyte maturation to enhance oocyte function and protect it from stress	Rodrigues et al. (2019)
	Porcine	In vitro	Oviductal fluid derived EVs (OEC‐EVs) in porcine significantly improved the concentration and distribution of cortical granules in oocytes. Furthermore, OECEVs also increased oocyte mitochondrial activity, reduced polyspermy and increased the IVF success rate	Fang et al. (2023)
	Bovine	In vitro	Follicular phase uterine EVs significantly increased the blastocyst rates of in vitro produced bovine embryos	Piibor et al. (2023)
	Porcine	In vitro	Enhanced in vitro oocyte maturation in pigs with follicular fluid exosomes is mediated by MiR‐339‐5p regulated ERK1/2 pathway through SFPQ	Han, Zhang, et al. (2024)
	Equine	In vitro	Follicular fluid derived EVs significantly enhanced cumulus expansion in both compacted and expanded cumulus–oocyte complexes, while viability increased in compacted group, but decreased in expanded group	Gabryś et al. (2024)
	Porcine	In vitro	EVs derived from porcine uterine fluid during the estrous phase carry bioactive molecules like glutathione, which help protect blastocysts from oxidative stress and enhance their development	Miura et al. (2024)
	Bovine	In vitro	Supplementation of the oocyte maturation media with follicular and ampullary fluid EVs positively influenced oocyte quality and enhanced in vitro maturation, fertilisation rates, and the TNFAIP6, HAS2, and GDF9 genes expression changes	Pakniyat et al. (2025)
EVs in improving oocyte maturation and embryo development	Bovine	In vitro	Exosomes in follicular fluid play important roles during oocyte maturation to enhance oocyte function and protect it from stress	Rodrigues et al. (2019)
	Porcine	In vitro	Oviductal fluid derived EVs (OEC‐EVs) in porcine significantly improved the concentration and distribution of cortical granules in oocytes. Furthermore, OECEVs also increased oocyte mitochondrial activity, reduced polyspermy and increased the IVF success rate	Fang et al. (2023)
	Bovine	In vitro	Follicular phase uterine EVs significantly increased the blastocyst rates of in vitro produced bovine embryos	Piibor et al. (2023)
	Porcine	In vitro	Enhanced in vitro oocyte maturation in pigs with follicular fluid exosomes is mediated by MiR‐339‐5p regulated ERK1/2 pathway through SFPQ	Han, Zhang, et al. (2024)
	Equine	In vitro	Follicular fluid derived EVs significantly enhanced cumulus expansion in both compacted and expanded cumulus–oocyte complexes, while viability increased in compacted group, but decreased in expanded group	Gabryś et al. (2024)
	Porcine	In vitro	EVs derived from porcine uterine fluid during the estrous phase carry bioactive molecules like glutathione, which help protect blastocysts from oxidative stress and enhance their development	Miura et al. (2024)
	Bovine	In vitro	Supplementation of the oocyte maturation media with follicular and ampullary fluid EVs positively influenced oocyte quality and enhanced in vitro maturation, fertilisation rates, and the changes in TNFAIP6, HAS2, and GDF9 gene expression	Pakniyat et al. (2025)
EVs in improving the process of in vitro fertilisation and embryo transfer	Bovine	In vitro and in vivo	Amniotic fluid‐derived microvesicles enhanced the hatching rate of in vitro‐produced embryos and improved pregnancy outcomes following embryo transfer	Lange‐Consiglio et al. (2020)

One of the primary applications of EVs in livestock reproduction is their use as fertility biomarkers. Identifying reliable biomarkers for spermatozoa quality (Rana et al. 2024), oocyte competence (Uzbekova et al. 2020), and embryo development (Dissanayake et al. 2020) is crucial for selecting the most viable and functional gametes and embryos for assisted reproductive technologies. Studies have shown that EVs isolated from seminal plasma contain microRNAs and proteins that correlate with sperm motility, viability, and fertilisation capacity (Barranco et al. 2019; Pal et al. 2025). Similarly, EVs from follicular fluid have been found to carry molecular signatures associated with oocyte maturation and developmental potential (Gabryś et al. 2022; Hung et al. 2015). Veterinarians and breeders can make informed decisions regarding breeding strategies and artificial insemination protocols by analysing the EV profiles in reproductive fluids.

EVs also have potential applications in sperm preservation and cryopreservation. Freezing and thawing procedures commonly used in artificial insemination can cause structural damage and reduce the viability of spermatozoa. Supplementing cryopreservation media with EVs derived from epididymal or seminal plasma has been shown to improve post‐thaw sperm motility and membrane integrity (Rodriguez‐martinez and Roca 2022). These vesicles provide protective effects by stabilising lipid membranes, reducing oxidative stress, and delivering key proteins involved in sperm function (Barranco et al. 2025). Enhancing sperm preservation techniques using EVs could lead to higher conception rates in artificial insemination programmes (Sawicki et al. 2024).

In embryo transfer programs and in in vitro fertilisation practices, EVs can be utilised to improve embryo culture conditions and implantation success. The early embryo relies on maternal signals from the oviduct and uterus to regulate gene expression and developmental processes. Co‐culturing embryos with EVs derived from oviductal and endometrial secretions has been shown to enhance blastocyst formation rates, reduce oxidative stress, and improve embryo survival (Han et al. 2025; Leal et al. 2022; Mazzarella et al. 2024; Piibor et al. 2024). These findings suggest that EVs could be used as bioactive additives in embryo culture media to mimic the physiological environment of the reproductive tract.

Another promising application of EVs in livestock reproduction is their potential use in reproductive immunomodulation. Pregnancy in mammals involves complex interactions between the maternal immune system and the developing fetus (Abu‐Raya et al. 2020). Inadequate immune tolerance to the embryo can lead to implantation failure or early pregnancy loss (Andreescu 2023). EVs secreted by the conceptus and maternal tissues help regulate immune responses by suppressing pro‐inflammatory cytokines and promoting regulatory T‐cell activity (Abeysinghe et al. 2023; Paktinat et al. 2021). Understanding how EVs contribute to maternal‐fetal immune tolerance could pave the way for developing therapeutic approaches to prevent pregnancy complications in livestock.

EVs may also play a role in improving the efficiency of cloning and somatic cell nuclear transfer. Cloning techniques are often associated with low success rates due to epigenetic abnormalities and improper reprogramming of the donor nucleus (Gouveia et al. 2020; Srirattana et al. 2022). Emerging evidence suggests that EVs derived from oocytes and early embryos contain epigenetic modifiers that may enhance nuclear reprogramming (Barrera et al. 2017; Estill et al. 2016). Researchers may be able to improve the developmental competence of cloned embryos and increase the efficiency of somatic cell nuclear transfer by incorporating EVs into cloning protocols.

## 9 Challenges and Limitations in EV Research

Despite the promising applications of EVs in reproductive medicine and livestock production, several challenges and limitations need to be addressed before their widespread implementation. One of the primary challenges in EV research is the standardisation of isolation and characterisation techniques. Various methods, including ultracentrifugation, size‐exclusion chromatography, and microfluidic‐based approaches, are used to isolate EVs from biological fluids (Welsh et al. 2024; Yakubovich et al. 2022). However, differences in isolation protocols can lead to inconsistencies in vesicle purity, yield, and functionality (Allelein et al. 2021; Ramirez et al. 2018). Developing standardised methodologies for EV isolation and characterisation is essential for ensuring reproducibility and comparability across studies.

Another limitation in EV research is the heterogeneity of vesicle populations. EVs are a diverse group of particles with varying sizes, cargo compositions, and biogenesis pathways. Distinguishing between exosomes, microvesicles, and apoptotic bodies remains a challenge due to overlapping size distributions and shared molecular markers (Allelein et al. 2021; Wang et al. 2025; Willms et al. 2018). Advances in single‐vesicle analysis techniques (Midekessa et al. 2021), such as high‐resolution flow cytometry (Barranco et al. 2024) and super‐resolution microscopy (Bağcı et al. 2022), may provide more precise methods for characterising EV subtypes.

The functional mechanisms of EVs in reproductive processes also remain incompletely understood. While studies have demonstrated the involvement of EVs in sperm maturation, oocyte competence, and embryo–maternal communication, the exact molecular pathways by which these vesicles exert their effects require further investigation (Dissanayake et al. 2024; Hasan et al. 2021; Hung et al. 2015; Muhandiram et al. 2024). Identifying the specific cargo molecules responsible for EV‐mediated signalling will be crucial for developing targeted therapeutic applications.

In clinical settings, the scalability and cost‐effectiveness of EV‐based therapies pose additional challenges (Adlerz et al. 2020; Ng et al. 2022). Large‐scale production of EVs for therapeutic use requires optimised cell culture conditions and efficient purification methods (Busatto et al. 2018; Kusuma et al. 2022; Liaqat et al. 2024). Furthermore, regulatory considerations regarding the safety, stability, and delivery of EV‐based treatments need to be addressed before they can be integrated into successful reproductive medicine applications and treatments (Wang et al. 2024).

## 10 Future Directions in Reproductive Biology and Physiology EV Research

As the field of EV research continues to evolve, several exciting avenues for future exploration have emerged. One promising direction is the development of engineered EVs for targeted reproductive therapies. Researchers can design vesicles with enhanced therapeutic properties by modifying EV cargo through genetic engineering or chemical modifications. For example, EVs engineered to carry specific microRNAs or proteins involved in sperm function could be used to treat male infertility. Similarly, EVs containing pro‐angiogenic factors may be utilised to improve placental vascularisation in cases of recurrent pregnancy loss.

Another important area of research is the use of EVs as drug delivery vehicles in reproductive medicine. EVs have inherent biocompatibility and the ability to cross biological barriers, making them ideal carriers for delivering drugs, hormones, or gene‐editing tools to reproductive tissues. Investigating the potential of EV‐based delivery systems for reproductive therapies could lead to innovative treatments for infertility, endometriosis, and other reproductive disorders.

EVs also hold promise for advancing non‐invasive diagnostics in reproductive health. The identification of EV‐derived biomarkers for conditions such as polycystic ovary syndrome, endometriosis, and recurrent pregnancy loss could provide clinicians with novel tools for early detection and personalised treatment strategies. Liquid biopsy approaches utilising EV analysis may revolutionise reproductive medicine by enabling real‐time monitoring of fertility status and pregnancy health.

In livestock reproduction, EV‐based approaches may contribute to sustainable breeding practices and genetic improvement programmes. Enhancing reproductive efficiency through EV‐mediated interventions could reduce the environmental impact of livestock production and improve food security. Further research into the role of EVs in gamete preservation and embryo transfer could optimise breeding strategies for economically important animal species.

## 11 Conclusions

EVs represent a rapidly expanding field of research with significant implications for reproductive biology, clinical fertility treatments, and livestock reproduction. Their ability to mediate intercellular communication, regulate reproductive processes, and serve as biomarkers for fertility assessment highlights their potential as transformative tools in reproductive medicine.

While challenges remain in standardising isolation techniques, characterising vesicle heterogeneity, and elucidating functional mechanisms, ongoing advancements in molecular biology, bioengineering, and nanotechnology are poised to address these limitations. The development of EV‐based diagnostics and therapeutics holds great promise for improving reproductive health outcomes in both humans and animals.

As the scientific community continues to unravel the complexities of EV biology, the integration of vesicle‐based approaches into clinical and agricultural settings will pave the way for innovative solutions in fertility management. The coming years are likely to witness groundbreaking discoveries in EV research, leading to novel applications that enhance reproductive success and advance the fields of reproductive medicine and livestock biotechnology.

## Author Contributions

**Alireza Fazeli:** conceptualized the study, conducted the literature review, wrote the original draft of the manuscript and acquired funding. **Kasun Godakumara:** contributed to literature review, contributed to write the original draft of the manuscript, provided critical insight and editing of this manuscript. **Suranga Kodithuwakku:** contributed to literature review, contributed to write the original draft of the manuscript, provided critical insight and editing of this manuscript. **Subhashini Muhandiram:** contributed to literature review, contributed to write the original draft of the manuscript, provided critical insight and editing of this manuscript.

## Conflicts of Interest

The authors declare no conflicts of interest.

## Data Availability

Data sharing not applicable to this article as no datasets were generated or analysed during the current study.
